# Minimally invasive versus open surgery among colorectal cancer patients

**DOI:** 10.6026/973206300211635

**Published:** 2025-06-30

**Authors:** Rahul Mathew, Kaushik J., Sailesh I.S Kumar, Shakthiyan Gopal, Ajeet Saoji, Priyanka Perumal, Shoraf P.

**Affiliations:** 1 Department of General Surgery, Govt. Stanley Medical College, Chennai, Tamil Nadu, India; 2 Department of Surgical Oncology, Rajiv Gandhi Cancer Institute & Research Centre, New Delhi, India; 3 Department of Surgery, University of Alabama, Birmingham, United States; 4 Department of Neuro Surgery, Sagar Hospital, Bengaluru, Karnataka, India; 5 Department of Community Medicine, N. K. P. Salve Institute Of Medical Sciences & Research Centre And Lata Mangeshkar Hospital, Digdoh, Nagpur, Maharashtra, India; 6 Department of General Surgery, SRM Medical College Hospital and Research Centre, Chengalpattu, Tamil Nadu, India; 7 Department of Community Medicine, Madha Medical College & Research Institute, Kovur, Chennai, Tamil Nadu, India

**Keywords:** Minimally invasive surgery (MIS), open surgery (OS), colorectal cancer, surgical outcomes, disease-free survival, postoperative recovery

## Abstract

Minimally Invasive Surgery has become an acceptable substitute for Open Surgery in the management of colorectal cancer, with the claimed benefits of reduced morbidity and quicker recovery. Therefore it is of interest to compare the clinical and oncological results of MIS and OS among 200 patients with colorectal cancer over five years. The patients were evaluated for operative time, complications, hospital stay and disease-free survival. MIS has been demonstrated to be linked to markedly reduced stays in hospital (6 vs. 10 days) and decreased complication rates (15% vs. 25%) versus Open Surgery with similar oncologic outcomes: clearance of margins, as well as five-year disease- free survival, p > 0.05. Thus, data support MIS as an effective and safe method within suitably chosen patients with cancer of the colon and rectum, with advantage at recovery duration and complications.

## Background:

Colorectal cancer is one of the leading causes of cancer morbidity and mortality world over, the treatment of which in localized disease is the cornerstone of surgery [1]. Open surgery is the classic method with outstanding oncologic outcomes [2]. Minimally invasive surgery like laparoscopic and robotic surgery gained favor as technology and knowledge improved [3]. The rest of the answer remains the same. MIS has certain advantages, such as minimal postoperative pain, reduced hospital stay, faster recovery and improved cosmetic outcome, at the cost of oncologic safety [4]. Despite all these advantages, its use in such complex cases-advanced stage tumor or comorbidity-is doubtful and its impact on long-term oncologic outcomes needs to be studied [5]. These comparative studies where MIS has been contrasted with OS have produced promising outcomes, but operative time, rate of complications and disease-free survival data diverge among studies [6]. Therefore, it is of interest to compare MIS and OS outcomes in clinical and oncologic results, operative factors, postoperative course and survival at long term in patients with colorectal cancer.

## Materials and Methods:

This was a comparative study over five years done in a tertiary care center with the enrollment of 200 patients with colorectal cancer who underwent open surgery (OS) or minimally invasive surgery (MIS). The patients were divided into two groups randomly: 100 underwent MIS and 100 underwent OS. Ethical approval was requested and informed consent was taken from all the participants. Inclusion criteria were patients 18 years and older with histologically proven colorectal cancer and no distant metastasis at presentation. Excluded was recurrent disease, prior abdominal surgery, or contraindication to MIS. Patient demographics, tumor size, location and stage and intraoperative variables like blood loss and operating time were recorded. Postoperative outcomes were complications, hospital stay and 30-day readmission. Oncologic outcomes recorded were clearance of surgical margins and five-year disease-free survival. Statistical testing on SPSS version 25 through chi-square tests and Kaplan-Meier analysis was performed to assess differences in outcomes between the groups. The p-value was set at <0.05.

## Results:

200 patients were enrolled in the study, of whom 100 received minimally invasive surgery (MIS) and 100 received open surgery (OS). The demographic and tumor profile were similar in both groups without any difference in mean age, gender ratio, or tumor stage. Table 1 (see PDF) presents a comparison of patients' demographic and tumor profile in MIS and OS groups based on similar baseline parameters in both groups. Table 2 (see PDF) highlights intraoperative outcomes, indicating that MIS was associated with significantly lower blood loss, but a slightly longer operative time compared to OS. Table 3 (see PDF) compares postoperative outcomes, demonstrating significantly shorter hospital stays and lower complication rates in the MIS group. Table 4 (see PDF) presents the oncological outcomes, showing comparable margin clearance and five-year disease-free survival rates between the two groups. Table 5 (see PDF) illustrates the distribution of postoperative complications, showing that MIS was associated with fewer wound infections and respiratory complications compared to OS. Table 6 (see PDF) compares the quality of life (QoL) scores between the two groups, with MIS patients reporting higher scores across physical, emotional and social domains at six months postoperatively. Table 7 (see PDF) presents the recurrence rates at five years, showing no significant difference between the MIS and OS groups, indicating comparable oncological safety. Table 8 (see PDF) highlights patient satisfaction scores, with MIS patients reporting higher satisfaction due to shorter recovery times and reduced pain levels. Table 9 (see PDF) compares the economic impact of the two surgical approaches, showing that MIS was associated with lower overall costs due to shorter hospital stays and fewer complications. Table 10 (see PDF) examines the return-to-work time among patients, with MIS patients resuming normal activities significantly earlier than those in the OS group.

The findings give detailed insights on comparative outcomes for minimally invasive surgery and open surgery for colorectal cancer patients. Table 1 (see PDF) provides demographic and tumor characteristics showing equal baseline parameters. Intraoperative outcome results show significantly low blood loss, however, the MIS group experienced operative times a bit longer than those with OS as in Table 2 (see PDF). Table 3 (see PDF) compares postoperative outcomes, showing that the MIS group had shorter hospital stays and fewer complications. Table 4 (see PDF) presents oncological outcomes, which are similar in terms of margin clearance and five-year disease-free survival rates between the two groups. Table 5 (see PDF) presents the distribution of postoperative complications, with fewer wound infections and respiratory complications in the MIS group. Table 6 (see PDF) compares QoL scores at six months postoperatively, with higher physical, emotional and social domain scores in the MIS group. Table 7 (see PDF) indicates no difference in the rates of recurrence at five years between the two groups and hence affirms the oncological safety of MIS. Table 8 (see PDF) presents better scores of patient satisfaction with MIS on account of short recovery periods and good pain control. Table 9 (see PDF) describes the economic analysis; the results suggest that overall, MIS is cheaper, having been associated with a shorter period of hospital stay and reduced complications. Table 10 (see PDF) shows a much earlier return-to-work time for the MIS patients, indicating faster recovery and better postoperative functionality. This summary shows all benefits of MIS: recovery, complications and economic impact, maintaining equal oncological outcomes with OS.

## Discussion:

This research presents the comparative outcomes of MIS and OS in colorectal cancer patients and highlights the clinical and cost advantages of MIS [7]. Results have determined that MIS had significantly decreased length of hospital stay, decreased postoperative complication rate and shorter recovery time compared to OS [7, 8]. For example, MIS patients had a mean hospital stay of 6 days versus 10 days in the OS group and a significantly reduced rate of complications at 15% versus 25% in OS (p < 0.05) [9, 10]. Oncological outcomes like surgical margin clearance and rates of five-year disease-free survival were comparable across the two groups, validating MIS oncological safety [11]. The rates of recurrence and rates of long-term survival were also not shown to have any statistically significant difference and further supported MIS as a proven alternative to OS [12].

Interestingly, patients in the MIS group had higher quality of life and satisfaction ratings, signifying the better recovery process and reduced postoperative morbidity [13]. Economic evaluation determined that MIS is associated with lower overall healthcare costs due to reduced hospital stays and complications. The shorter return-to-work periods of MIS patients, where 70% returned to activity within 30 days, also highlight the functional benefits of this approach [14]. The results validate the overall application of MIS in colorectal cancer surgery from the literature. The study however indicates patient selection and surgical skill to guarantee optimal results. Long-term oncological outcomes, MIS technology innovation and measures to increase access to minimally invasive procedures are topics that require priority in research.

## Conclusion:

Minimally invasive surgery (MIS) has clear clinical and economic advantages over open surgery (OS) in patients with colorectal cancer. MIS has lower postoperative complications, faster recovery, shorter hospital stay, and higher patient satisfaction without detrimental oncologic safety on the basis of equivalent surgical margins and five-year disease-free survival. The findings recommend expanded utilization of MIS in combination with advances in surgical technology and residency training to optimize colorectal cancer therapy.

## References

[1] Feng Q (2022). Lancet Gastroenterol Hepatol..

[2] Grosek J (2021). Radiol Oncol..

[3] Minciuna C.E (2023). Chirurgia (Bucur)..

[4] Ozair A (2022). Surg Endosc..

[5] Kampman S.L (2023). Int J Colorectal Dis..

[6] Villano A.M (2020). J Surg Res..

[7] Guidolin K (2021). Dis Colon Rectum..

[8] Yeo H.L (2016). J Surg Res..

[9] Zhang X (2016). Surg Endosc..

[10] Babaei M (2016). Medicine (Baltimore)..

[11] Behman R (2023). Ann Surg..

[12] Bizzoca C (2021). Cancers (Basel)..

[13] Prete F.P (2018). Ann Surg..

[14] Carpenter E.L (2023). Surg Endosc..

